# A Question of Economy for Hospital Boards

**Published:** 1912-11-16

**Authors:** 


### PROBLEMS IN ADMINISTRATIVE MEDICINE.

(Carefully thought-out Contributions to this Section will be welcome.)

### A Question of Economy for Hospital Boards.

From time to time a degree of friction arises between hospital boards and hospital medical officers over the question of the cost of drugs and extras ordered for patients. A case in point was alluded to recently in these columns, where a doctor had ordered strawberries out of season at a time when they could only be procured for seven shillings and sixpence a pound (The Hospital, October 5, 1912, p. 4). As a general rule the governors of a hospital are so absolutely in the hands of their medical staff over this and kindred matters that they are very loath to interfere in any way with the unfettered discretion of the doctors as to what shall be done for the needs of the individual patients. Generally speaking, it is certainly the wiser plan for boards to trust their medical staff; and, if it should appear that any member or members of the latter are failing to practise economy over drugs, dressings, diet, or anything else, it is usually more effective to deal with the matter quite unofficially through the tactful offices of the medical representative of the staff upon the lay board.

The broad rule having thus been stated, we proceed to draw attention to a not uncommon case in which it may be violated with good results. The point we are about to raise is one which affects many hospitals, and in respect of which very great pecuniary savings can be effected without the slightest loss of efficiency in the service of the hospitals concerned. To make quite clear the whole matter at issue it is necessary to state certain simple propositions which are not so well known, even to the medical profession, as they should be.

### The Price of Anæsthetics.

Every hospital uses ether and chloroform in considerable quantities; and the cost of these anæsthetics, which are, of course, essential in any hospital where operative surgery is undertaken, forms a not inconsiderable item in the total annual drug bill. Ether can be made, and is commercially made, from two sources: methylated spirit and rectified spirit. Chloroform can be and is made from three sources: methylated spirit, rectified spirit, and acetone. Now rectified spirit pays a very heavy duty to the Revenue, while acetone and methylated spirit do not. It therefore happens that ether and chloroform manufactured from rectified spirit cost, roughly, three times (rather more in the case of ether, rather less in the case of chloroform) as much as the same drugs made from the non-dutiable materials. Chloroform made from acetone costs very slightly less than that made from methylated spirit; and this disparity may be increased if anything comes of a new process, recently described, by which the cost of acetone will be reduced to less than half what it is at present.

Is there any difference in the final products prepared by these different processes? Is one superior to another in safety, efficiency, after-effects, or any other way? The question is for experts to answer; and, unfortunately, they are divided in opinion over it. The new edition,* just published, of Sir Frederic Hewitt's text-book of anæsthetics (which we hope to review on a later occasion) contains some important information to which attention may be drawn. To begin with, Sir Frederic himself has always maintained that there is no difference whatever in the actions and effects of the drugs however prepared, provided that they are properly purified. In this opinion he is supported by Dr. Dudley Buxton and other authorities almost of equal eminence with himself; but the contention is denied by a good many other anæsthetists whose opinions are entitled to respect. Further, Sir Frederic is backed up by Sir Edward Thorpe, the eminent chemist, who avers that no analyst can detect the source of manufacture of a purified specimen of ether or chloroform.

### Sir Frederic's Referendum.

To ascertain the general current of feeling among London anæsthetists a circular was sent round to every holder of a hospital appointment (in that specialty) in London on a certain date. The replies are analysed in Sir Frederic's text-book, and they show a division of opinion which is certainly remarkable. Into the actual figures and statistics of this form of Referendum there is no need to plunge. Suffice it that opinion is fairly evenly divided: those who maintain that the ether and chloroform prepared from dutiable spirit are superior to the same drugs manufactured from the other sources mentioned above, are about balanced numerically by those who hold, with Sir Frederic, that there is no appreciable difference at all. In addition, there are a small number who assign a definite *inferiority* to the rectified spirit products.

It may be said, therefore, that whatever difference there may be is minute; and that there is no certainty that any such difference exists at all except in the imagination. It would seem reasonable to infer that the ordinary principle of buying in the cheapest market would decide the medical profession in favour of the non-dutiable products, which cost about a third of the other sort. In hospitals, at least, this consideration might be expected to weigh. In point of fact, many hospitals still adhere to the expensive rectified spirit articles. This conservatism may be justified by those members of the medical staff who administer the anæsthetics if they honestly believe in the superiority of the more costly sorts; but it becomes a very serious question whether governing boards should indulge this predilection in view of the slender basis of solid support which it appears to have. In a certain small hospital of some sixty beds or so, with which we are acquainted, the annual bill for anæsthetics has been cut down to less than half of what it

* *Anæsthetics and their Administration.* By Sir F. W. Hewitt, Anæsthetist to H.M. the King. Fourth Edition. 1912. Macmillan and Co. 15s. net.

was four years ago, by the simple process of ordering the methylated ether and chloroform instead of the rectified spirit products; and this, in spite of a steady increase in the number of operations performed each year and in the average time occupied by each.

In this particular case the change was made with the full consent of the medical staff: indeed, at their own suggestion. But the question is one which it is well within the province of a lay board to take in hand, when once the simple main facts of the situation have been comprehended. Backed up by the authority of Sir Frederic Hewitt—the admitted expert of experts in this branch of practice—we do not suppose that any medical staff would oppose a recommendation in favour of the cheaper kinds of anæsthetic pressed upon them by a governing board. We should be extremely sorry to promote by word or deed any cause of offence or friction between hospital managers and their professional staffs; but we do unhesitatingly express the opinion that, in the event of such opposition to the change, a lay board would be justified in insisting upon it, even if it led to resignations amongst the honorary medical staff. We estimate the saving which can be thus effected to be between ten shillings and a sovereign per (surgical) bed per annum; in a large hospital this soon mounts up.

It is within our knowledge that at a certain well-known lying-in hospital anæsthetics are administered to the patients only when some abnormal complication renders the performance of an obstetric operation necessary. Subscriptions are being solicited on behalf of this hospital on the express grounds that chloroform and ether are so expensive that in the ordinary cases of labour they cannot be afforded; and that an increased income will enable poor mothers to be granted the boon of anæsthesia in their travail. We raise no objection whatever to this method of exciting the generosity of the pitiful; any plea for money in order to promote greater efficiency in a hospital commands our sympathy. But we are bound to observe that the quantity of chloroform required to relieve the ordinary patient in a normal confinement would not on the average exceed an ounce, or two ounces at the outside; and that the cost of an ounce of acetone chloroform to a hospital would not be more than three halfpence. Even rectified spirit can be obtained at not much more than fourpence or fivepence for this quantity. We trust, therefore, that when the authorities of the institution we have in mind decide that they can afford to anæsthetise patients for the latter part of the second stage of normal labour, they they will buy the more economical kind of anæsthetic. And we trust that the same lesson of practical businesslike economy, without any diminution of efficiency, will be laid to heart both by hospital governing boards and by their honorary and resident medical staffs.

